# Small RNA transcriptomes of mangroves evolve adaptively in extreme environments

**DOI:** 10.1038/srep27551

**Published:** 2016-06-09

**Authors:** Ming Wen, Xingqin Lin, Munan Xie, Yushuai Wang, Xu Shen, Zhongqi Liufu, Chung-I Wu, Suhua Shi, Tian Tang

**Affiliations:** 1 State Key Laboratory of Biocontrol, Guangdong Provincial Key Laboratory of Plant Resources and Key Laboratory of Biodiversity Dynamics and Conservation of Guangdong Higher Education Institutes, School of Life Sciences, Sun Yat-sen University, Guangzhou 510275, Guangdong, China; 2 Department of Ecology and Evolution, University of Chicago, 1101 E. 57th Street, Chicago, IL, 60637, USA

## Abstract

MicroRNAs (miRNAs) and endogenous small interfering RNAs (siRNAs) are key players in plant stress responses. Here, we present the sRNA transcriptomes of mangroves *Bruguiera gymnorrhiza* and *Kandelia candel.* Comparative computational analyses and target predictions revealed that mangroves exhibit distinct sRNA regulatory networks that differ from those of glycophytes. A total of 32 known and three novel miRNA families were identified. Conserved and mangrove-specific miRNA targets were predicted; the latter were widely involved in stress responses. The known miRNAs showed differential expression between the mangroves and glycophytes, reminiscent of the adaptive stress-responsive changes in *Arabidopsis*. *B. gymnorrhiza* possessed highly abundant but less conserved *TAS3 trans*-acting siRNAs (tasiRNAs) in addition to tasiR-ARFs, with expanded potential targets. Our results indicate that the evolutionary alteration of sRNA expression levels and the rewiring of sRNA-regulatory networks are important mechanisms underlying stress adaptation. We also identified sRNAs that are involved in salt and/or drought tolerance and nutrient homeostasis as possible contributors to mangrove success in stressful environments.

Plants have evolved remarkable ways to sense and cope with various environmental stresses. Being adapted to challenging environments, extremophile plants represent a valuable resource for understanding the evolutionary processes of stress adaptation and its underlying mechanisms^1^,^2^. Whereas the mechanisms of stress adaptation have been largely studied at the genomic and transcriptional levels, the importance of posttranscriptional regulation has been emphasized with the identification of stress-responsive small RNAs (sRNAs) in plants^3^,^4^.

MicroRNAs (miRNAs) and small interfering RNAs (siRNAs) are two major groups of sRNAs in plants^3^,^4^,^5^. MiRNAs are generated from RNA polymerase II (Pol II)-transcribed single-stranded primary miRNAs (Pri-miRNAs) and are then processed by DICER-LIKE 1 (DCL1) into mature products that guide target mRNA cleavage and/or translation inhibition. Unlike miRNAs, siRNAs are derived from RNA-dependent RNA polymerase (RdRP)-generated double-stranded RNAs and mediate DNA methylation or trigger target mRNA cleavage^5^. Plant miRNAs preferentially target transcripts encoding transcriptional factors and play pivotal roles in developmental and phase transitions, nutrient homeostasis and stress responses^5^,^6^. Plant miRNAs can also initiate the biogenesis of *trans*-acting siRNAs (tasiRNAs) through cleavage of *TAS* transcripts^7^,^8^,^9^. The vast majority of miRNAs that are involved in plant growth and development show altered expression profiles in response to biotic and abiotic stresses, indicating that miRNAs mediate stress-responsive developmental modulation^3^,^4^. Despite the importance of miRNAs in plant stress tolerance, the miRNA repertoire has only been reported for limited extremophiles, such as *Thellungiella salsuginea*^10^.

Mangroves are a group of woody plants that grow in tropical and sub-tropical intertidal zones and estuaries^11^. As extremophiles, mangroves have evolved various morphological, physiological and reproductive adaptations to cope with abiotic stresses, such as high salinity, anoxia and nutrient-poor substrates^11^. There has been increasing interest in studying mangroves to better understand plant adaptations to extreme environments^11^,^12^,^13^. A recent study demonstrated that the two mangroves *Heritiera littoralis* and *Rhizophora mangle* share distinct transcriptome profiles, differing from those of stress-sensitive plants^13^. This finding makes us wonder whether sRNA profiles have been embodied in the unique mangrove “lifestyle”. If so, how could the distinct mangrove sRNA profiles shed light on the molecular mechanisms underlying plant stress adaptations? Currently, only red sea mangrove (*Avicennia marina*) miRNAs have been identified by high-throughput sequencing^14^. Information on sRNA expression profiles is scarce for most mangrove species. No attempt has been made to compare the mangrove sRNA repertoire with that of stress-sensitive plants.

Here, we deep sequenced the sRNA libraries from the leaf and flower tissues of *Bruguiera gymnorrhiza* and *Kandelia candel*, two viviparous and salt-excluding mangrove species. We demonstrated the differential expression of miRNAs between mangroves and glycophytes, which resembled the adaptive responses of these miRNAs under various abiotic stresses. MiRNA target prediction and the expanded features of putative tasiRNAs reveal the rewiring of the sRNA regulatory networks in mangroves.

## Results

### Deep sequencing analysis of small RNA libraries from *B. gymnorrhiza* and *K. candel*

**Figure 1:**
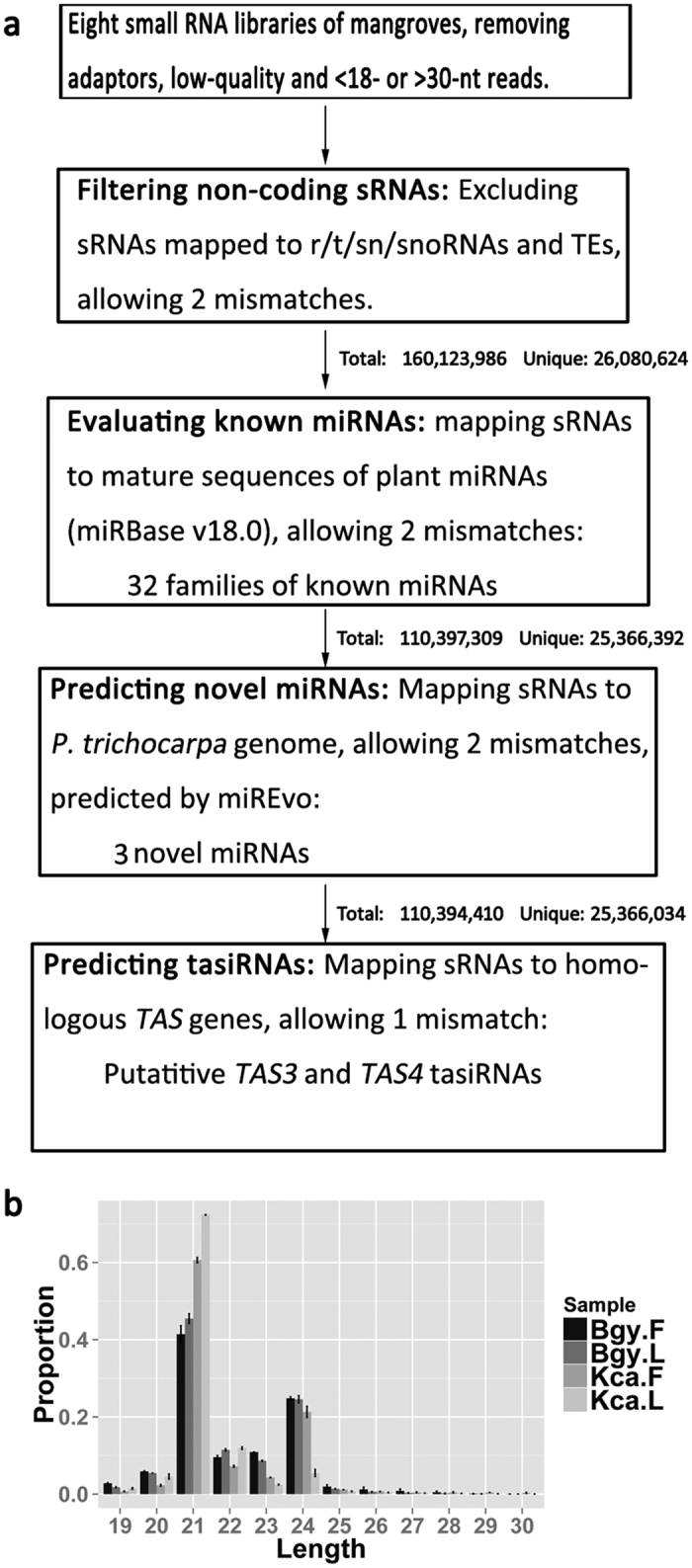
The characterization of small RNA (sRNA) populations in *Bruguiera gymnorrhiza* and *Kandelia candel*. (**a**) Pipeline for the identification of miRNAs and tasiRNAs from mangrove sRNA libraries. The number of candidates and the resultant sRNAs after each step are indicated. (**b**) The sRNA size distributions from the libraries of the flower (F) and leaf (L) tissues of *B. gymnorrhiza* (Bgy) and *K. candel* (Kca). The error bar indicates the SD (n = 2).

Small RNA libraries were constructed from the leaves and flowers of *B. gymnorrhiza* and *K. candel,* each with two biological replicates. The workflow of the sRNA annotation is shown in Fig. 1a. Approximately 180 million clean reads were obtained from eight libraries, corresponding to 27.5 million unique reads (Table S1). After excluding reads matching the structural non-coding RNAs and repetitive sequences, the remaining 89.2% of total reads were used to evaluate known miRNAs and to predict novel miRNAs. As shown in Fig. 1b, the 21-nt sRNAs, accounting for 41.4–71.2% of the total reads, were the most abundant in all of the libraries, followed by 24- and 22-nt sRNAs (5.5–24.8% and 7.2–11.9%, respectively). In plants, 21-nt sRNAs are usually canonical miRNAs, whereas 24-nt sRNAs consist mainly of sRNAs that are associated with repeats and TEs^5^. *K. candel* had more 24-nt sRNAs in the flower buds (21.3%) than in the leaves (5.5%) and had a greater proportion of 21-nt sRNAs than *B. gymnorrhiza* (66.5% vs. 51.0%, Fig. 1b), indicating differential expression of sRNAs between tissues and species of these mangroves.

### Identification of the known and novel miRNAs in mangroves

**Figure 2:**
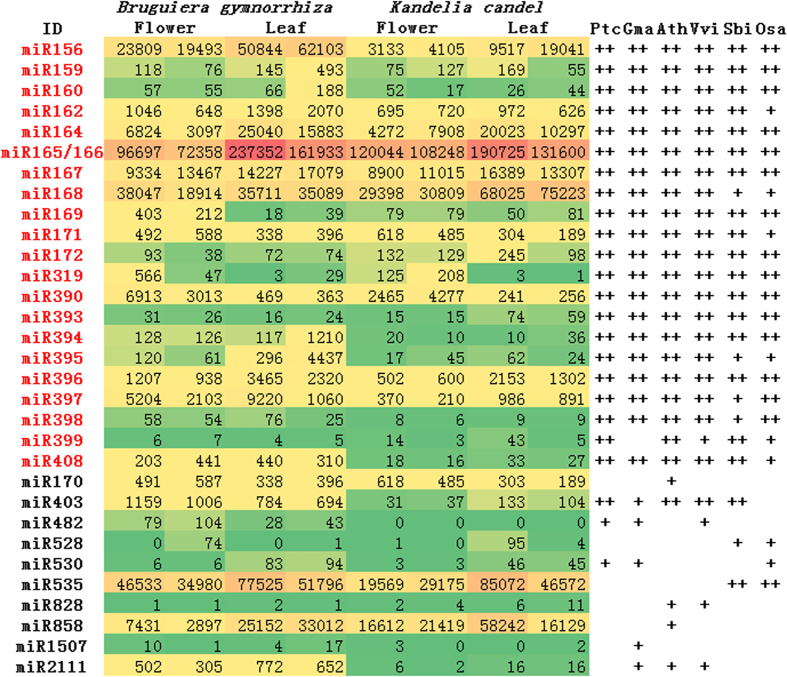
Read abundance and sequence conservation of known miRNA families in the flower and leaf tissues of *B. gymnorrhiza* and *K. candel.* Numbers indicate the RPM (reads per million) of the representative mature miRNAs for the individual miRNA families in each sample. The sequence conservation of the 32 known miRNA families in *B. gymnorrhiza* and/or *K. candel* was checked in *Arabidopsis thaliana* (Ath), *Glycine max* (Gma), *Populus trichocarpa* (Ptc), *Vitis vinifera* (Vvi), *Sorghum bicolor* (Sbi), and *Oryza sativa* (Osa). The symbols indicate the following: ++, at least one mature sequence in the mangrove miRNA families is identical to a sequence in another species; +, miRNA sequences are present with mismatches in other species; and −, miRNA sequences are absent in other species. The conserved and less-conserved miRNA families are indicated in red and black, respectively.

We refer to the known miRNAs from 22 families that are conserved in both monocots and eudicots as conserved miRNAs^15^, whereas those from miRNA families reported in either monocot or eudicot lineages are referred to as less-conserved miRNAs. By mapping short reads to miRNA precursors registered in miRBase (Release 18), we identified 32 known miRNA families collectively from *B. gymnorrhiza* and *K. candel*, including 255 distinct mature miRNAs from 21 conserved and 11 less-conserved families (Fig. 2). MiR827 was the only undetectable conserved miRNA in both species. The expression level of each mature miRNA was measured as reads per million miRNA-matched reads (RPM) (Table S3). The most conserved mature miRNA for each miRNA family was then used for further analyses (see Materials and Methods). As shown in Fig. 2, the conserved miRNAs were generally more abundant than the less-conserved miRNAs, with miR165/166 the most abundant in all libraries (on average, 139,200 RPM), followed by miR168, miR156, miR167 and miR164 (>9,000 RPM per library. Tissue-preferential expression was also observed for miR156 and miR530 in the leaves and miR390 in the flowers of mangroves (Fig. 2).

After removing the short reads matching known miRNAs, the remaining sRNAs were used to predict novel miRNAs by a modified miRDeep2 algorithm^16^ with the genome of *P. trichocarpa*^17^ as a mapping reference. According to the Angiosperm Phylogeny Group III system^18^, *P. trichocarpa* is most closely related to Rhizophoraceae among the species with available whole genomes. The predicted novel miRNAs exhibited a canonical stem-loop structure (Fig. S2a), and the presence of the homologous transcripts of these predicted novel miRNA precursors was detected by homology search (blastn, e < 6 × 10^−9^) against the *R. apiculata* transcriptome (Fig. S2b). Two of the novel miRNAs were further supported by the presence of miRNA^*^ (Table 1) and PCR cloning of the genomic loci in *B. gymnorrhiza* and *K. candel* (Fig. S2b). Moreover, the averaged MFEI of the novel miRNAs was 1.73 ± 0.34, consistent with a previous observation that miRNAs exhibit a higher MFEI value than tRNAs (0.64), rRNAs (0.59), and mRNAs (0.65)^19^. The predicted novel miRNAs were expressed at much lower levels than the known miRNAs and often exhibited species-preferential expression. As shown in Table 1, bgy-miR1001 is more abundant in *B. gymnorrhiza* than in *K. candel* (146 vs. 50 reads per library), whereas the expression of bgy-miR1002 is eight times higher in *K. candel* than in *B. gymnorrhiza* (197 vs. 24 reads pre library).

### Conserved and specific targets of mangrove miRNAs

Plant miRNAs recognize their target mRNAs through perfect or near perfect base-pairing, mainly in the coding region^5^. Based on a set of 10,938 unigenes of *B. gymnorrhiza*^12^, we predicted 81 target genes for 98 mature miRNAs from 22 known families and one novel miRNA family using psRNAtarget^20^. The lack of known predicted targets for some conserved miRNAs is likely due to the incomplete transcriptome information available for *B. gymnorrhiza*. The predicted targets of the known miRNAs were frequently involved in metabolism, membrane transport and response to stimuli (Fig. S3), suggesting diverse roles of miRNA regulation in mangrove development and stress responses. The novel bgy-miR1001 was predicted to target BP940488, a casein kinase II subunit gene (*CKB*) that is well-conserved in regulating eukaryotic circadian clocks^21^.

**Figure 3:**
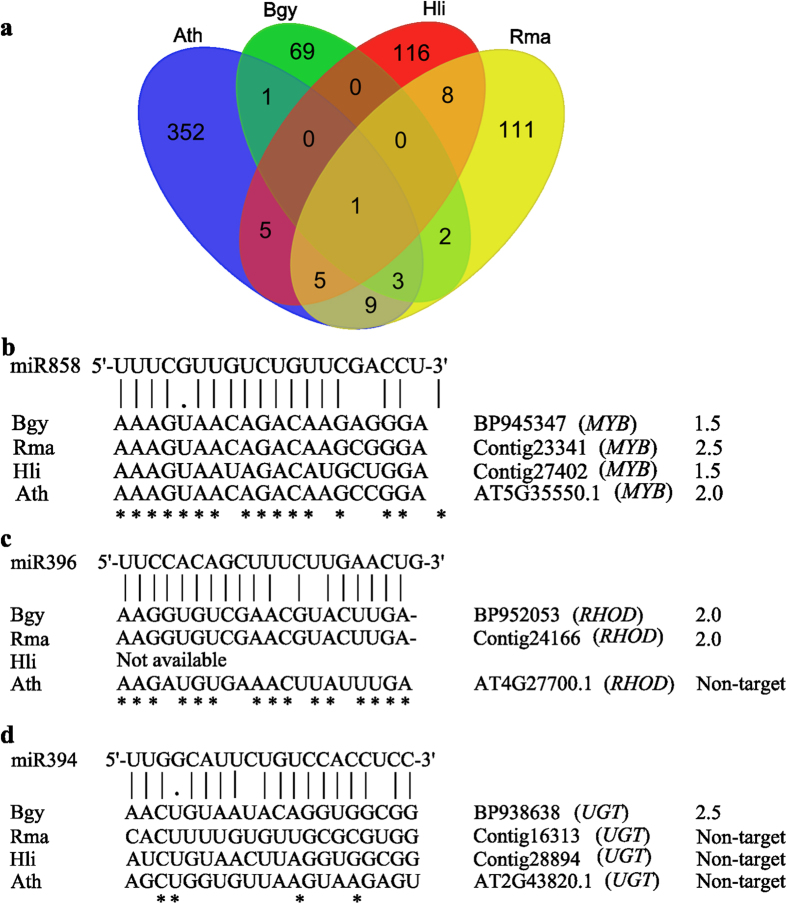
Conserved and unconserved targets of mangrove miRNAs. (**a**) Venn diagram describing the overlap of the predicted miRNA targets among *B. gymnorrhiza* (Bgy)*, R. mangle* (Rma), *H. littoralis* (Hli) and *A. thaliana* (Ath). (**b**) A target of miR858 that is conserved among mangroves and *Arabidopsis*. (**c**) A target of miR396 that is conserved among mangroves. (**d**) A target of miR394 that is unique to *B. gymnorrhiza*. Numbers indicate the complementarity score between the miRNAs and their target transcript. The higher the scores, the more detrimental the mismatches for miRNA function. In the alignments, the vertical lines indicate matches between the miRNA and target, the missing lines indicate mismatches, and the dots indicate G:U wobble pairs. The asterisks indicate identical residues among all four species.

To assess the conserved and specific miRNA regulations among phylogenetically diverse mangrove species, we predicted targets for the same collection of known miRNAs in two additional mangroves — *R. mangle* and *H. littoralis* — based on their transcriptome data^22^ and using *Arabidopsis* as a reference. The predicted miRNA targets had different levels of sequence conservation, of which twenty-four miRNA-target pairs were shared by *Arabidopsis* and at least one mangrove species (Fig. 3a). These conserved miRNA targets were enriched for genes encoding transcriptional factors, such as *SBP* (miR156), *NAC* domain protein and *UXS* gene (miR164), *ARF* (miR167), *AP2* (miR172), *TCP2* (miR319), *AFB* (miR393), F-box gene (miR394), *APS* (miR395), *bHLH* (miR396) and *MYBs* (miR828/miR858, Fig. 3b), consistent with the results of the miRNA target prediction in red sea mangrove^14^. In addition to transcriptional factors, conserved miRNA targets also include genes with well-known functions in stress responses and nutrient deprivation, such as nuclear factor Y (*NF-Ys*, miR169) in drought stress^23^, Cu/Zn *SODs* (miR398) in copper deprivation and oxidative stress^24^ and ATP sulfurylase (*APS*, miR395) in sulfate deprivation^25^. However, we failed to identify some well-characterized miRNA targets — such as the miR399 target phosphate 2 (*PHO2*), which maintains plant phosphate (Pi) homeostasis^26^ — due to the lack of homologous sequences in mangroves.

Ten predicted targets common in at least two mangrove species but absent in *Arabidopsis* were classified as mangrove-specific miRNA targets (Fig. 3a and Table S4). These targets included rhodanese/cell cycle control phosphatase (miR396, Fig. 3c), which is involved in leaf senescence and defense responses^27^; *LETM1*-like protein (miR162), photosystem II reaction center protein L (*PSBL*, miR171), ATP synthase gamma chain 1 (*ATPC1*, miR172), and NAD(P)-binding Rossmann-fold superfamily protein (*UXE*, miR172), which are involved in photosynthesis and/or photorespiration^28^; transducin/WD40 repeat-like superfamily protein (miR156) and P-loop containing nucleoside triphosphate hydrolase superfamily protein (NTPase, miR172), which are involved in diverse developmental processes and stress signaling^29^; and the heat shock protein *ARG1*-like 1 (*ARL1*, miR156), TCP-1/cpn60 chaperonin family protein (miR172) and Nudix hydrolase homolog gene (*NUDT*, miR528), which act as key regulators of stress responses^30^. The conserved miRNAs acquired new targets involved in stress tolerance in mangroves.

In addition to mangrove-specific miRNA targets, conserved miRNAs also acquired species-specific regulatory relationships. For example, miR394 regulates the auxin signaling F-box protein in diverse plants^4^ but was predicted to target UDP-glucosyltransferase (*UGT*)-encoding gene in *B. gymnorrhiza* (Fig. 3d). The loss of function of the *Arabidopsis UGT71C1* gene is known to improve resistance to oxidative damage^31^. Using 5′ RACE, we validated the conserved miRNA target Cu/Zn *SOD1* of miR398 and the mangrove-specific miRNA target rhodanese/cell cycle control phosphatase of miR396 (Fig. 3c) in *B. gymnorrhiza* (Fig. S4).

### Differential expression of known miRNAs between mangroves and glycophytes

We now address whether mangroves exhibit differential miRNA expression from stress-sensitive plants. The abundance of known miRNAs was compared between the two mangroves (*B. gymnorrhiza* and *K. candel*) and four glycophyte species (*Arabidopsis*, *Populus*, grape and sorghum), for which sRNA sequencing data are available for leaves and/or flowers (Table S2). We did not include *A. marina* in this analysis because sRNAs were sequenced from pooled tissues in this species^14^.

**Figure 4:**
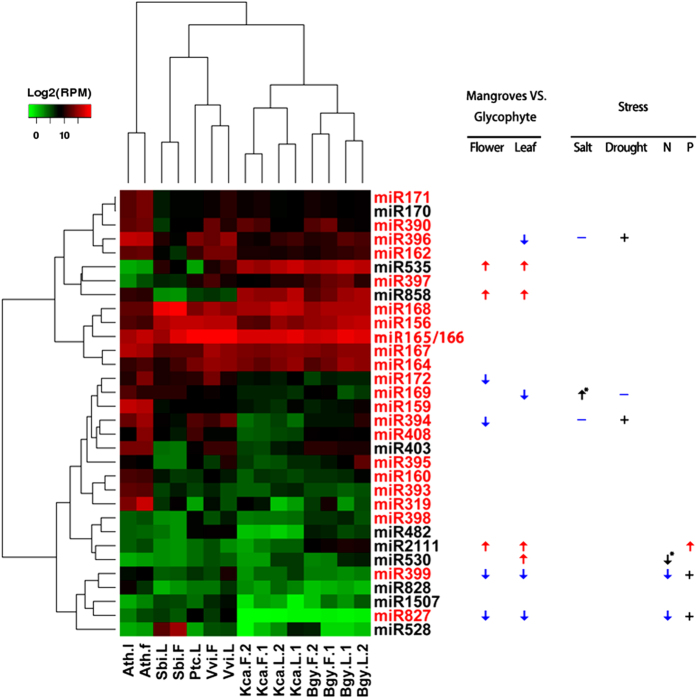
The differential expression of known miRNAs between mangroves and glycophytes. The heatmap is log2 (RPM) of 32 known miRNAs in the flower (F) and leaf (L) tissues of mangroves (*B. gymnorrhiza* (Bgy) and *K. candel* (Kca)) and glycophytes (*A. thaliana* (Ath), *P. trichocarpa (*Ptc), *V. vinifera* (Vvi), and *S. bicolor (*Sbi)). The conserved and less-conserved miRNA families are indicated in red and black, respectively. MiR827 was included in the differential expression analysis despite its absence in mangroves. The mangrove-excessive and mangrove-deficient miRNAs are indicated with upward and downward arrows in red and blue, respectively. The function and differential expression of these miRNAs under salt, drought, (−)N and (−)P stress are indicated as +, positive regulators; −, negative regulators; ↑, upregulation; and ↓, downregulation. The data were adapted from studies in *Arabidopsis* combined with two data points from rice, which are indicated with an asterisk. For each miRNA, its function or expression under a certain stress that matched its differential expression between mangroves and glycophytes is indicated in the same color as for the excessive or deficient expression in mangroves.

Hierarchical clustering analysis clustered known miRNAs from mangroves and glycophytes into two distinct clades (Fig. 4). Nine of the 32 known miRNAs were found by edgeR^32^ to be differently expressed between mangroves and glycophytes, with an adjusted p-value < 0.05 and a fold change >2 in at least one tissue (Fig. 4). The differentially expressed miRNAs were further classified as mangrove-excessive miRNAs (miR530, miR535, miR858 and miR2111) or mangrove-deficient miRNAs (miR399, miR169, miR396, miR172 and miR394) according to their expression levels relative to those of glycophytes (Fig. 4). The excess of miR535 and miR858 in mangroves was remarkable in both tissues (Fig. 4). miR535 is upregulated by low temperature in the Phalaenopsis orchid^33^, whereas miR858 together with miR828 triggers the production of *TAS4* tasiRNAs by repressing *MYBs*^34^. The remaining differentially expressed miRNAs are responsive to either salt/drought stress (miR169, miR394 and miR396) or nutrient deprivation (miR399, miR2111 and miR530), except miR172, which regulates flowering time and flowering patterning^6^.

miR169, miR394 and miR396, which act as negative regulators of plant drought or salt stress tolerance in *Arabidopsis*^23^,^35^,^36^, showed deficient expression (30- to 215-fold decrease on average) in the flowers and leaves of mangroves (Fig. 4). The downregulation of miR169 upon drought stress confers enhanced drought tolerance through the de-repression of *NY-F* in *Arabidopsis*^23^, whereas miR169 is upregulated by drought and salt in rice^37^,^38^. MiR394 suppresses salt tolerance while simultaneously promoting drought tolerance in *Arabidopsis* through the repression of *LCR*^35^. Similarly, the overexpression of miR396 decreases salt tolerance but confers drought tolerance in *Arabidopsis*^36^. Among miRNAs that participate in nutrient homeostasis, miR399 was mangrove-deficient, whereas miR2111 and miR530 were mangrove-excessive (Fig. 4). MiR399, miR2111 and the absent miR827 in mangroves (Fig. 2) are specifically induced in response to phosphate deprivation in *Arabidopsis*^26^,^39^,^40^. MiR399 represses *PHO2* — a repressor for nitrate-dependent Pi uptake^26^,^39^ — and is repressed upon N starvation in *Arabidopsis*^40^,^41^ owing to the antagonistic interaction between Pi and nitrate^40^. In contrast, miR399 is induced by N starvation, whereas miR530 is repressed by N starvation in rice^42^. Taken together, the differential expression of miRNAs between mangroves and glycophytes is consistent with their adaptive responses upon various stresses in *Arabidopsis*, although the stress-responsive miRNA profiles can vary under different stresses and between different species.

We further examined the tissue-preferential expression of miRNAs in mangroves using a generalized linear model. Ten miRNAs were differentially expressed between tissues in mangroves (adjusted p-value < 0.05 and fold change >2 in at least one species), including eight leaf-biased miRNAs (miR156, miR164, miR165/166, miR395, miR396, miR530, miR535 and miR858) and two flower-biased miRNAs (miR319 and miR390) (Fig. S5). The majority of these miRNAs exhibited divergent tissue-preferential expression between mangroves and glycophytes, such as miR165/166 with leaf-biased expression in *B. gymnorrhiza* but flower-biased expression in *Arabidopsis* and grape (Fig. S5). In contrast, the tissue-preferential expression of miR535, miR319 and miR390 was conserved among mangroves and glycophytes (Fig. S5). To confirm the results of RNA-seq, we conducted qRT-PCR of 12 miRNAs and validated the differential expression patterns between mangroves and glycophytes and/or between the tissues of mangroves in three-fourths of the comparisons (Fig. S6).

### Trans-acting siRNAs and their targets in mangroves

In plants, three miRNAs are known to participate in the biogenesis of tasiRNAs. The miR173-*TAS1*/*TAS2* pathway has only been reported in *Arabidopsis*^7^, whereas the miR390-*TAS3* and miR828-*TAS4* pathways are conserved in the plant kingdom and eudicots, respectively^8^,^9^. To identify tasiRNAs in mangroves, we first identified *BgTAS3* (BP947370) and *BgTAS4* (BP945347, *MYB*) by BLASTx against *B. gymnorrhiza* unigenes (e < 0.001) and then mapped short reads from *B. gymnorrhiza* to *BgTAS3* or *BgTAS4* using Bowtie^43^, allowing one mismatch. Approximately 85% of the short reads matching *BgTAS3* were 21 nt in length, which is the canonical length for tasiRNAs, whereas the short reads matching *BgTAS4* had an even distribution, with 21-nt reads accounting for only 49% of the matched reads (Figs S7 and S8).

**Figure 5:**
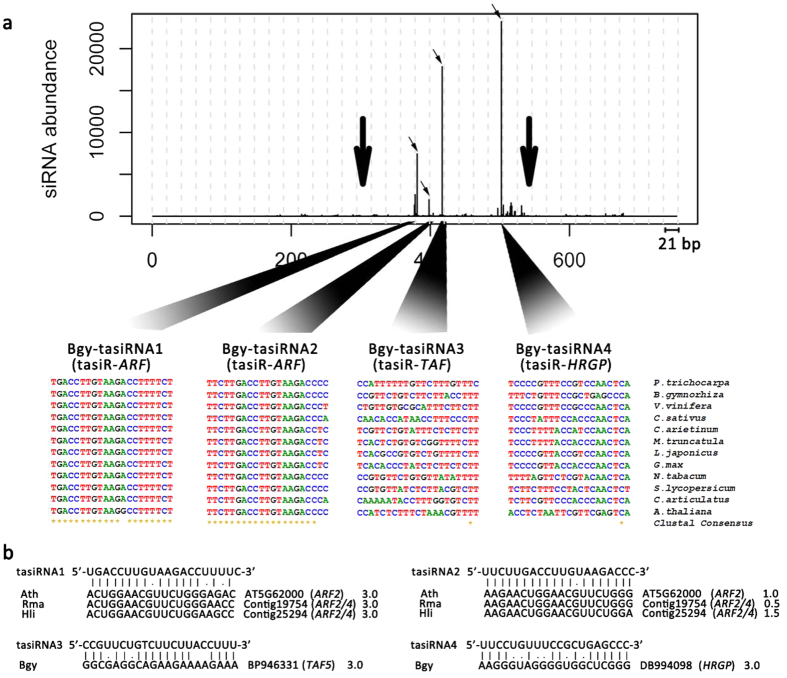
Putative *TAS3* tasiRNAs in *B. gymnorrhiza*. (**a**) Schematic diagram of siRNA biogenesis in the *BgTAS3* transcripts (BP947370) in a flower bud library of *B. gymnorrhiza.* Plots for the other libraries are given in Fig. S5. The vertical axis of the box indicates the averaged abundance of the 21-nt read counts. The horizontal axis of the box is plotted with dashed lines with a scale of 21 nt. The target site of miR390 is indicated with a bold arrow, and the four most abundant tasiRNAs are indicated with narrow arrows. Below the box is the sequence conservation for each type of tasiRNA among plant species, including *A. thaliana* (AT3G17185)*, B. gymnorrhiza* (BP947370)*, Curio articulatus* (JN692259)*, Cicer arietinum* (XR_189283)*, Cucumis sativus* (XR_181103)*, Glycine max* (XR_418344)*, Lotus japonicus* (AK338955)*, Medicago truncatula* (AC186679)*, Nicotiana tabacum* (FJ804751)*, P. trichocarpa* (XM_006378492)*, Solanum lycopersicum* (JX047545) and *V. vinifera* (XM_002273850). The asterisks indicate identical residues among all species. (**b**) Sequence pairings between the tasiRNAs and their target sites. The vertical lines indicate matches, the missing lines indicate mismatches, and the dots indicate G:U wobble pairs.

We detected 21-nt phased siRNAs (phasiRNAs) — 1.6-fold more abundant in leaves than in flowers — derived from *BgTAS3* (Figs 5 and S7), which were considered as putative *TAS3* tasiRNAs. In *Arabidopsis,* the generation of *TAS3* tasiRNAs is triggered by a “two-hit” model of miR390-directed cleavage^8^. The two target sites of miR390 were both conserved in the *BgTAS3* transcripts, from which Bgy-tasiRNA1 and Bgy-tasiRNA2 were derived (Fig. 5). Two unconserved regions of *BgTAS3* produced three-fold more abundant phased-tasiRNA registers, Bgy-tasiRNA3 and Bgy-tasiRNA4 (Fig. 5), indicating that the *TAS3* tasiRNAs biogenesis pathway has evolved unique features in *B. gymnorrhiza*.

Both Bgy-tasiRNA1 and Bgy-tasiRNA2 were predicted to target *ARF* genes (*ARF2* and *ARF4*). The miR390-*TAS3*-*ARF* pathway is conserved in higher plants^44^, in which tasiRNA-*ARFs* function collectively with miR165/166 in the specification of organ polarity and morphogenesis^45^. In contrast, Bgy-tasiRNA3 and Bgy-tasiRNA4 were predicted to target the TATA-binding protein-associated factor 5 (*TAF5*) and a gene encoding a hydroxyproline-rich glycoprotein (*HRGP*). *TAF5* is involved in Pol II transcription initiation, histone acetylation and chromatin modification^46^, whereas *HRGP* transcripts accumulate in response to various biotic and abiotic stresses as a defense mechanism because hydroxyproline-rich glycoproteins are important structural components of plant cell walls^47^.

The putative *TAS4* tasiRNAs, i.e., 21-nt phasiRNAs derived from *BgTAS4,* were in low abundance and had low reproducibility (Fig. S8). Considering the low miR828 level and high miR858 level in *B. gymnorrhiza* (Fig. 2), the biogenesis of *TAS4* tasiRNA in this species is likely triggered by miR828. Moreover, 21-nt sRNAs were barely mapped to the annotated *MYB* genes in *B. gymnorrhiza* (less than ten reads per library per site), suggesting that the biogenesis of miR828-mediated *TAS4* tasiRNAs is not as active as it is in other plants, such as *Arabidopsis*^34^ and apple^44^.

## Discussion

The convergent evolution of mangroves under common extreme environments occurs at the transcriptome level^13^. Here, we demonstrate that the mangroves *B. gymnorrhiza* and *K. candel* differ substantially from glycophytes in their miRNA profiles (Fig. 4). Two-thirds of the mangrove-excessive and mangrove-deficient miRNAs respond to salt (drought) stress and nutrient deprivation (Fig. 4). Most importantly, altered expression of these miRNAs largely resembles the miRNA expression changes that confer enhanced stress tolerance in *Arabidopsis* (Fig. 4), suggesting that evolutionary changes in miRNA expression may contribute to mangrove adaptations to high-salinity and low-fertility environments. Convergent evolution of miRNA expression may have also occurred in mangroves, as evidenced by the absence of miR827 consistently observed in this study and in *A. marina*^14^.

Mangrove soils are characterized by high salinity and extreme deficiencies in N and P^48^. High salinity prevents arbuscular mycorrhizal (AM) fungi from living in mangrove soils^49^, which hinders the uptake of nutrients such as P^50^. The anoxic conditions and high organic matter content result in a high denitrification rate, depleting the nitrate and nitrite pools in mangrove soils^11^,^48^. It is therefore not surprising that mangroves have reprogrammed expression profiles of the miRNAs that are associated with salt tolerance and nutrient uptake. Consistent with our findings, a recent study proposed that the differential expression of miRNAs might contribute to the survival of the intertidal snail *Littorina littoralis* during natural freezing or anoxia exposure^51^, indicating that alteration of miRNA expression is a prevalent phenomenon in the adaptation to environmental stresses.

The adverse interactions of multiple environmental factors in mangrove swamps may have shaped the mangrove miRNA profiles in a complex manner through the crosstalk of overlapping pathways^3^,^4^. The contrasting expression patterns of miR399 (deficiency) and miR2111 (excess) in mangroves are a result of crosstalk that reconciles the negative interactions between Pi and nitrate. In *Arabidopsis,* miR399 and miR827 maintain nitrate-dependent phosphate homeostasis^26^,^39^, whereas miR2111 responds exclusively to Pi starvation^40^. The deficiency in miR399, coincident with the absence of miR827 in mangroves, is consistent with a scenario of severe N starvation overwhelming Pi starvation. To cope with multiple stresses, the miRNAs in mangroves may play the role of canalization in buffering gene expression against fluctuating intertidal environments^52^. Moreover, the tissue-specific differential expression and the divergent tissue-preferential expression of miRNAs between mangroves and glycophytes (Figs 4 and S5) suggest that attenuation of miRNA expression is important for developmental modulation during mangrove adaptation to extreme environments.

In addition to expressional changes, miRNAs in mangroves have evolved an expanded target repertoire (Fig. 3). For example, miR396 and miR394 were predicted to recruit the new targets *RHOD* and *UGT* (Fig. 3c,d), which are involved in defense responses and oxidative resistance, respectively^27^,^31^. MiR396 recently gained the target *HaWRKY6,* which confers high-temperature protection in the sunflower *Helianthus annuus*^53^, indicating that acquiring novel miRNA regulation is an important mechanism underlying stress resistance in extremophiles. It is therefore likely that the predicted novel miRNA regulations in mangroves play a role in adaptive development and metabolism responses to environmental stresses.

The rewiring of tasiRNA pathways may also contribute to mangrove stress adaptations. *B. gymnorrhiza* possesses two highly abundant *TAS3* tasiRNAs with potential new targets *TAF5* and *HRGP*, which participate in essential cellular process (Fig. 5). We suspect that the novel tasiRNA regulations unique to mangroves might enable the stability of gene expression and/or chromatin structure, reconciling the conflicting demands of growth and stress resistance in the avoidance of overreaction to long-term stress exposure. In support of this hypothesis, the mangrove *Ceriops tagal* maintains transcriptional homeostasis in saline environments^54^. In the future, it will be interesting to validate the potentially rewired sRNA regulatory relationships and to elucidate their biological roles in adaptive stress responses.

In summary, mangroves differ from glycophytes with respect to their expression profiles and the regulatory relationships of miRNAs and tasiRNAs. Optimization of miRNA expression and rewiring of sRNA regulatory networks are important mechanisms underlying adaptation to extreme environments.

## Methods

### Plant material

Plant material from *K. candel* and *B. gymnorrhiza* was collected in a field near Dongzhai Harbor, Hainan, China. Samples of the leaves and flower buds were harvested and immediately stored in RNAlater (Applied Biosystems/Ambion, Austin, TX, USA) until RNA extraction. Two biological replicates were prepared for each tissue per species, resulting in eight samples in total.

### RNA isolation and small RNA sequencing

Total RNA was extracted using a modified CTAB method^55^ and was evaluated using an Agilent 21100 Bioanalyzer (Agilent Technologies). Small RNA libraries were prepared using the standard protocols of the Illumina Small RNA Sample Prep Kit and were sequenced using an Illumina Genome Analyzer (Illumina, San Diego, CA, USA) at BGI (Shenzhen, China).

### Computational analysis of the sequencing data

The pipeline of sRNA sequencing data processing is illustrated in Fig. 1a. After trimming off adaptor sequences and removing the low-quality and low-complexity reads, clean reads ranging from 19 to 30 nt were aligned against Rfam (Rfam 11.0)^56^ and RepeatMasker database (RepBase 12.0)^57^ using Bowtie (options: -f -v 2 -a –best –strata)^43^. Reads matching structural non-coding RNAs, such as rRNA, tRNA, snRNA, snoRNA, and repetitive sequences, were removed from further analysis.

### Identification of known and novel miRNAs

Plant mature miRNAs and their precursors were retrieved from miRBase (Release 18)^58^. Short reads from each sRNA library were mapped to known miRNA hairpins using Bowtie^43^, allowing up to two mismatches. Reads matching each of the mature miRNA sequences registered in miRBase were counted. Mature miRNAs mapped with more than 10 raw reads in both biological replicates were retained for further analysis. The abundance of each mature miRNA was normalized by the total number of miRNA reads in a given library and was scaled to reads per million (RPM). After excluding reads matching known miRNAs, the remaining reads were mapped to the genome of *Populus trichocarpa* (v1.1, ftp://ftp.ncbi.nih.gov/genomes/Populus_trichocarpa/) using Bowtie^43^ to predict novel miRNAs. miREvo was also used to predict novel miRNAs^16^.

### MiRNA target prediction and sequence analysis

A total of 11,997 ESTs of *B. gymnorrhiza* were retrieved from a previous microarray analysis (Supplemental Table 5 of ref. 13) and clustered into 10,938 unigenes using USEARCH^59^, requiring more than 85% identity. The transcriptomes of *H. littoralis* and *R. mangle* were downloaded from http://mangrove.illinois.edu22. Based on these data, psRNAtarget^20^ was used to predict the miRNA targets in each mangrove species using the default settings. Sequence conservation of the predicted miRNA targets was compared across the three mangrove species and *Arabidopsis* using the *Arabidopsis* gene models as references. The between-species overlaps of conserved targets were analyzed using a 4-way Venn diagram web tool (http://bioinformatics.psb.ugent.be/webtools/Venn/). Gene ontology (GO) annotations were conducted using the AgBase GOanna package^60^ with a BLASTx search against the “Plant” database.

### MiRNA differential expression analysis

The sRNA sequencing data from the leaves and/or flowers of *Arabidopsis thaliana*, *Populus trichocarpa, Vitis vinifera* and *Sorghum bicolor* were retrieved from a public database (Table S2) and were processed as illustrated in Fig. 1a. The expression levels of the known miRNAs in each library were calculated and normalized as RPM, as described above for the mangrove data. To conduct the cross-species differential expression analysis, the most conserved mature miRNA across all of the surveyed species was identified for each miRNA family (Table S3) and was used as the representative mature miRNA for further analysis. When more than one mature miRNA exhibited the same degree of conservation, the most abundant was chosen if it was on average >1.5-fold more abundant than the other(s), or an arbitrary one was selected if all of the conserved mature miRNAs were expressed at comparable levels. Differential expression analyses of the representative mature miRNAs were conducted 1) between mangroves and glycophytes for each tissue separately and 2) between different tissues of mangroves using edgeR^32^. For the former, a one-way layout design was used to construct the generalized linear model (GLM); for the latter, a nested design (eight samples for two species and two tissues) was used. In all of the comparisons, miRNAs with a fold-change ≥2 and an adjusted p-value ≤ 0.01 were considered differentially expressed.

### Identification and target prediction of tasiRNAs

To identify putative tasiRNAs, *TAS* transcripts were identified by BLASTx^61^ search against the *B. gymnorrhiza* unigenes with a cut-off of e < 0.001; short reads from the *B. gymnorrhiza* sRNA libraries were then mapped to the EST sequences of *BgTAS3* (BP947370) and *BgTAS4* (BP945347) using Bowtie^43^, allowing one mismatch. The targets of *TAS3* tasiRNAs in *B. gymnorrhiza* were predicted using psRNAtarget^20^ with the default settings.

### Quantitative real-time PCR of miRNAs

Stem-loop real-time PCR^62^ was used for miRNA quantification as described previously^63^. Briefly, total RNA was reverse-transcribed using TaqMan MicroRNA Reverse Transcription Kit (Invitrogen) and stem loop primers. The first-strand cDNA was then used as a template for real-time PCR in a 20 μL reaction mixture containing 0.1 μM Universal ProbeLibrary Probe #21 (Roche), 0.4 μL of 1 μM miRNA-specific forward and universal reverse primer. All reactions were run in triplicate on a LightCycler 480 instrument system (Roche, Germany). miRNA expression was normalized against that of 5.8S rRNA. The primers used for the reverse transcription and real-time PCR reactions are listed in Table S5.

### Detection of miRNA cleavage products using 5′ RACE

The total RNA (1 μg) from leaves of *B. gymnorrhiza* was used to synthesize 5′ RACE-ready cDNA with universal primer mix (UPM) and the SMARTer® RACE 5′/3′ Kit (Clontech, Palo Alto, CA, USA) according to the manufacturer’s instructions. The gene-specific primers were designed based on the ESTs of the potential miRNA target genes in *B. gymnorrhiza* (Table S5). Amplicons corresponding to the size of the expected cleavage products were gel-purified, cloned into PMD18-T vector (Takara, Otsu, Japan) and sequenced.

## Additional Information

**Accession codes**: The sequencing data were deposited in the NCBI Gene Expression Omnibus (GEO, http://www.ncbi.nlm.nih.gov/geo/) under the accession number GSE56442.

**How to cite this article**: Wen, M. *et al.* Small RNA transcriptomes of mangroves evolve adaptively in extreme environments. *Sci. Rep.* **6**, 27551; doi: 10.1038/srep27551 (2016).

## Supplementary Material

Supplementary Information

Supplementary Table S3

Supplementary Table S4

## Figures and Tables

**Table 1:** Predicted novel miRNAs in *Bruguiera gymnorrhiza* and *Kandelia candel*.

miRNA	Mature (5′-3′)	Length (nt)	Count								MFE/MFEI	Star
			Bgy.F.1	Bgy.F.2	Bgy.L.1	Bgy.L.2	Kca.F.1	Kca.F.2	Kca.L.1	Kca. L.2		
bgy-miR1001	TATCGAGTAGTAATTCAGGCA	21	179	156	134	116	42	48	46	66	−21.8/1.82	No
bgy-miR1002	TGTGCTTATGGACGGTCTTAT	21	20	15	27	33	171	135	275	207	−23.2/1.36	Yes
bgy-miR1003	TGAGATCCGATAGTATGGTAG	21	33	53	48	46	37	53	12	15	−22.3/2.03	Yes

The abbreviations are defined as follows: Bgy, *B. gymnorrhiza*; Kca, *K. candel*; F, flower; L, leaf; 1/2, the number of biological repeats; MFE, the minimum free energy of hybridization between the mature miRNA and star sequences; and MFEI, minimal folding free energy index.
