# Supplementary material for: Small RNA transcriptomes of mangroves evolve adaptively in extreme environments
Source: Sci Rep. 2016 Jun 9;6:27551. doi: 10.1038/srep27551 (PMC4899726; doi:10.1038/srep27551)
Supplement: Supplementary Information [file srep27551-s1.doc]

Article title: **Small RNA transcriptomes of mangroves evolve adaptively in extreme environments**

Authors: **Ming Wen, Xingqin Lin, Munan Xie, Yushuai Wang, Xu Shen, Zhongqi Liufu, Chung-I Wu, Suhua Shi and Tian Tang**

**Fig. S1. Pairwised correlation of miRNA expression levels between libraries.** The number embedded in each upper triangle box is the Pearson correlation coefficient. Abbreviations: RPM, Reads Per Million; Bgy, *B. gymnorhiza*; Kca, *K. candel*; F, flower buds; L, leaf; 1/2, biological repeats.


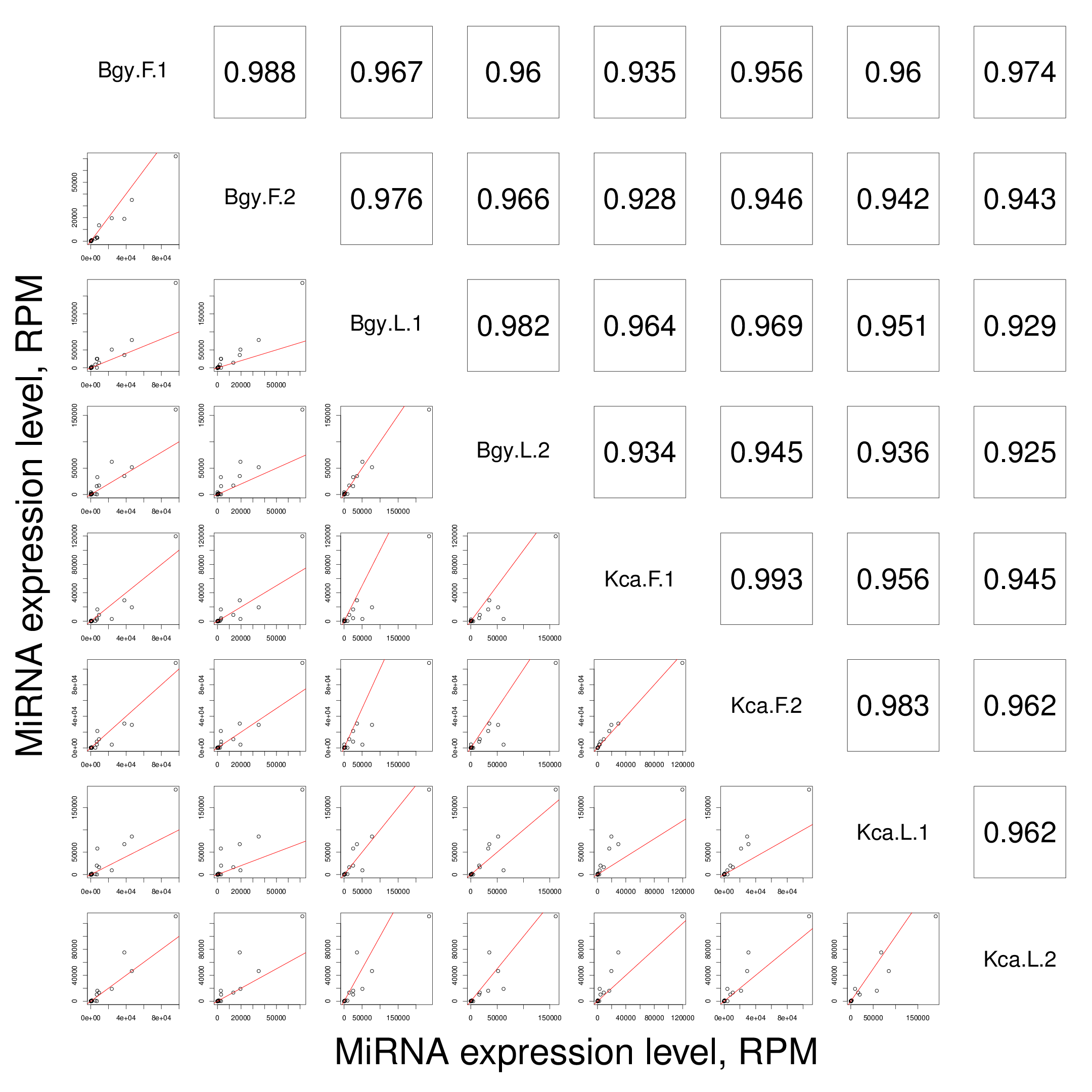


Fig. S1

**Fig. S2 Predicted novel miRNAs in mangroves.** (a) Hairpin sequences and secondary structures of the novel mangroves miRNAs. Mature sequences are given in upper case; Number inside the brackets denote as minimum free energy. (b) Sequence alignment of predicted new miRNA loci in *R. apiculata, B. gymnorrhiza* or *K. candel.*

**
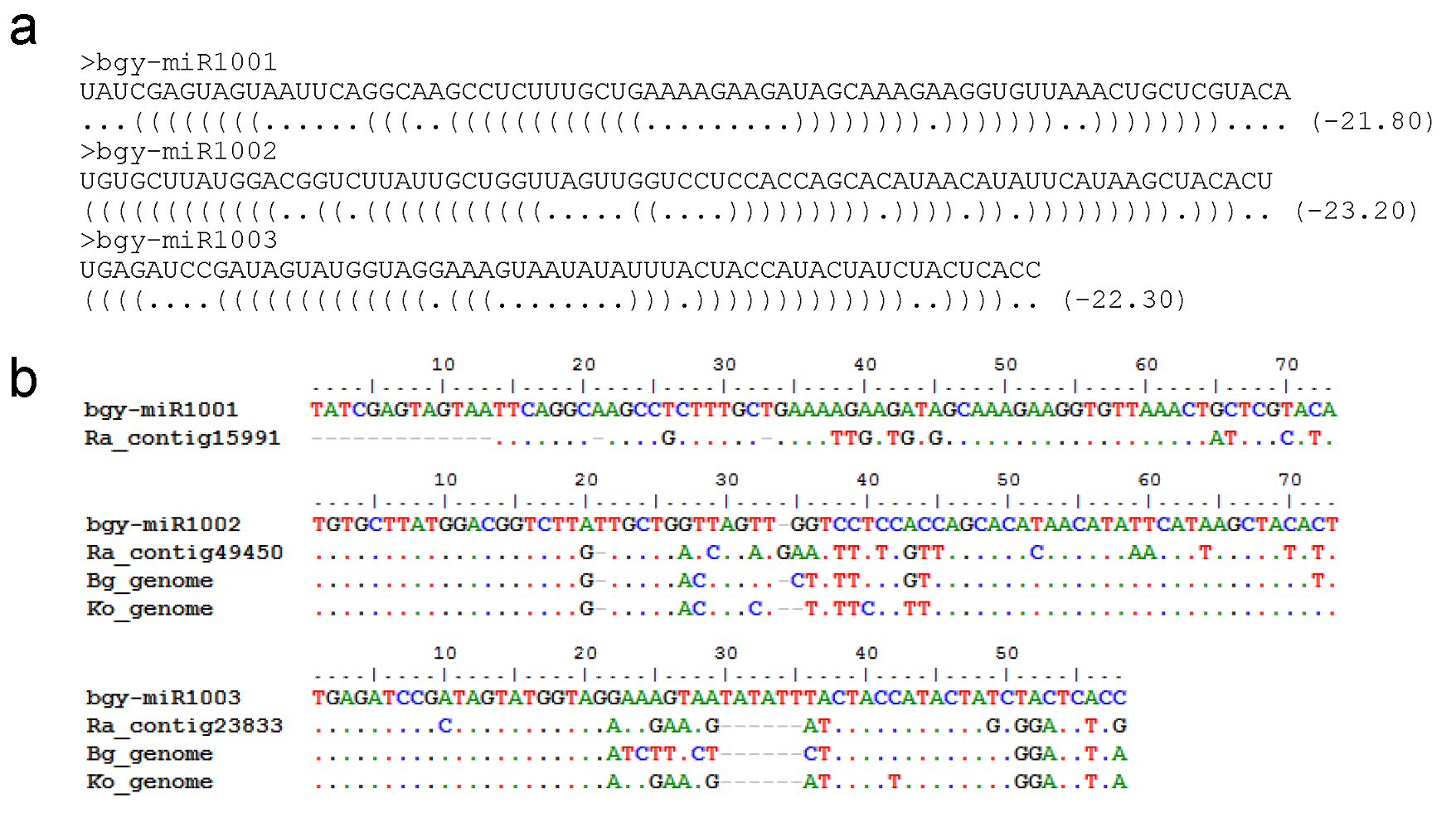
**

Fig. S2

**Fig. S3. Functional classification of predicted miRNA targets in *B. gymnorhiza* based on GO annotations.** GOSlim-plant categories based on the ontology of biological process were used.


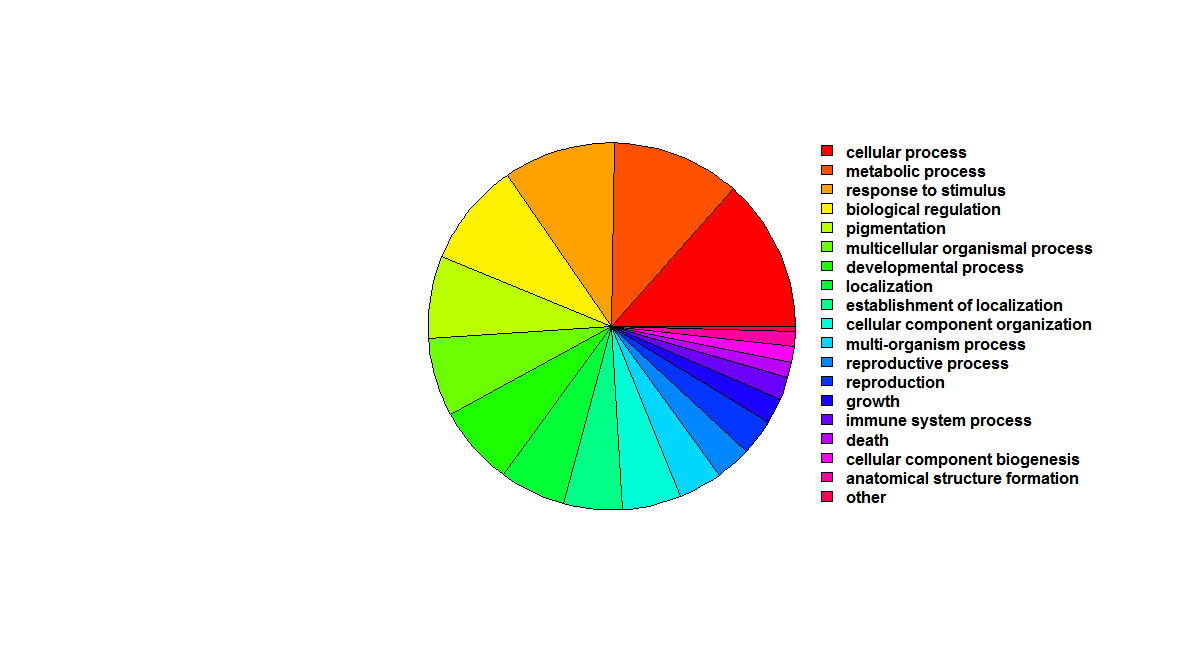


Fig. S3

**Fig. S4. Experimental validation of the predicted miRNA target genes of mangrove Brugiera gymnorrhiza.**  MiRNA-guided sites were identified by 5’ RACE-PCR. PCR products were cloned and sequenced. Arrows indicate mapped cleavage positions with the frequency amongst clones sequenced. Target gene sequences are shown on top of the miRNA sequences.


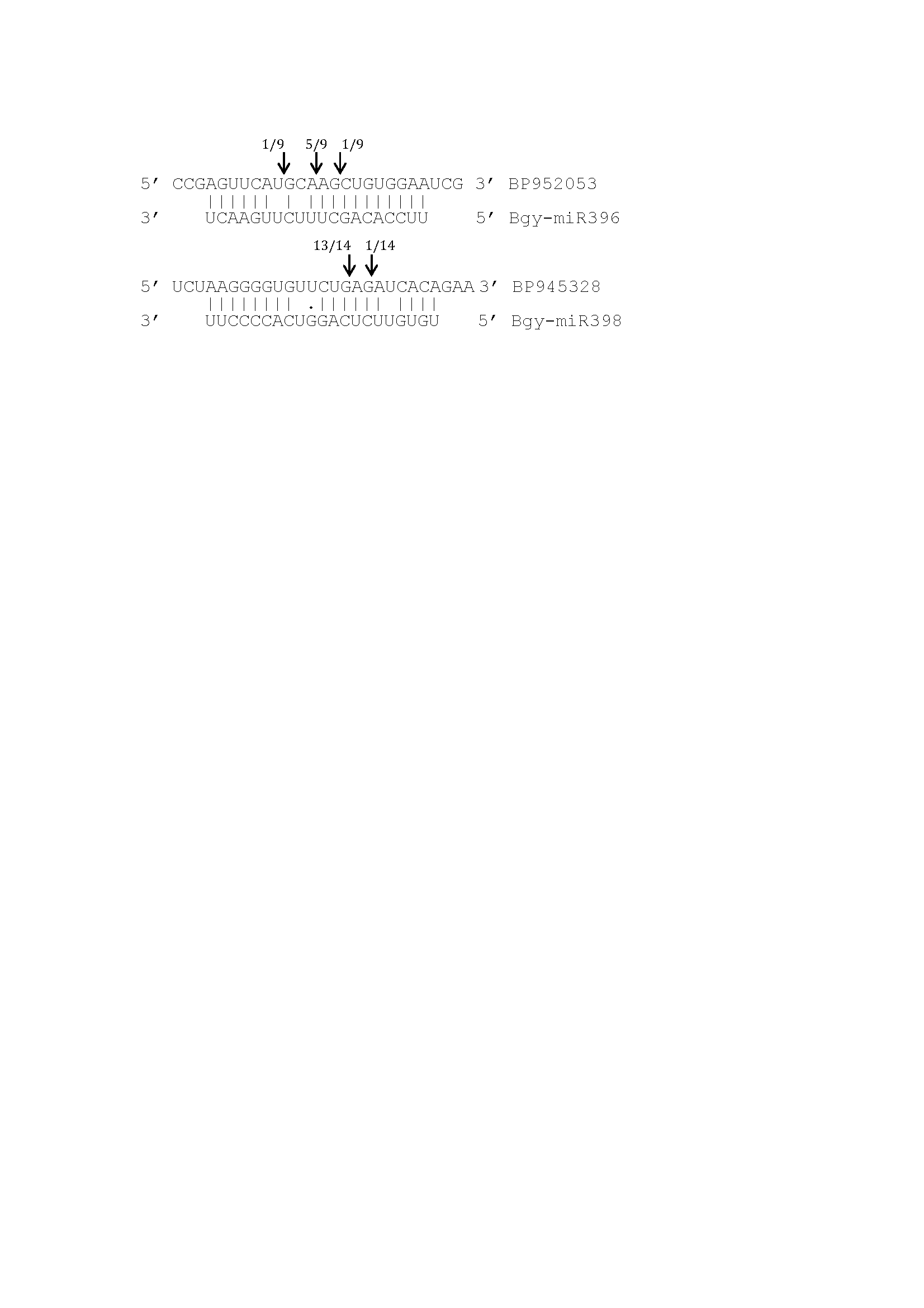


Fig. S4

**Fig. S5. Tissue-preferential miRNA expression in mangroves compared with glycophytes.** Log2 fold changes of miRNA expression between flowers and leaves were plotted for eight miRNAs that exhibited significant tissue-preferential expression in mangroves. Red and black bars represent mangrove and glycophyte species, including *B. gymnorhiza* (Bgy) and *K. candel* (Kca), and *A. thaliana* (Ath), *V. vinifera* (Vvi), and *S. bicolour* (Sbi), respectively. The error bar indicates SE (n=2). NA, not available. Asterisks indicate fold-changes greater than 2 and FDR <= 0.05.


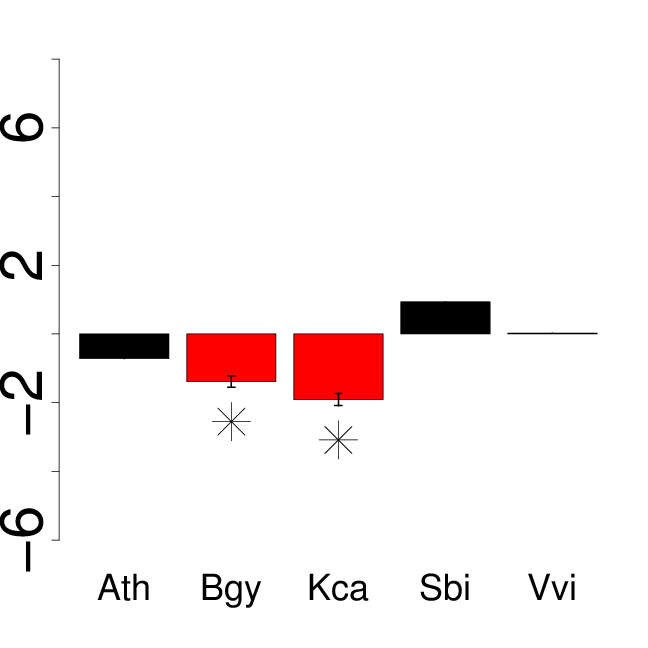

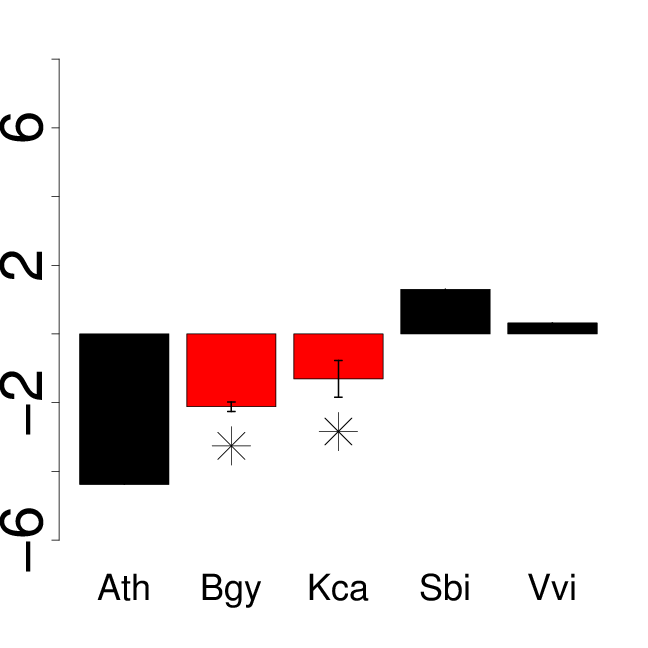

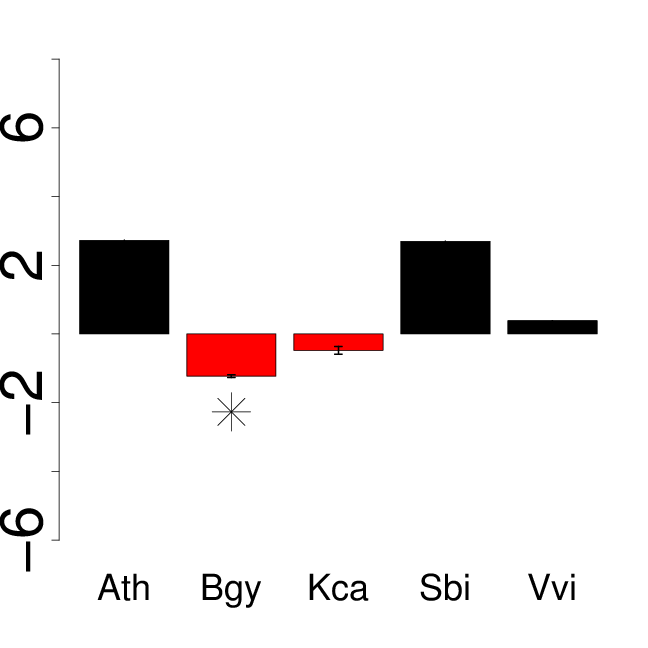

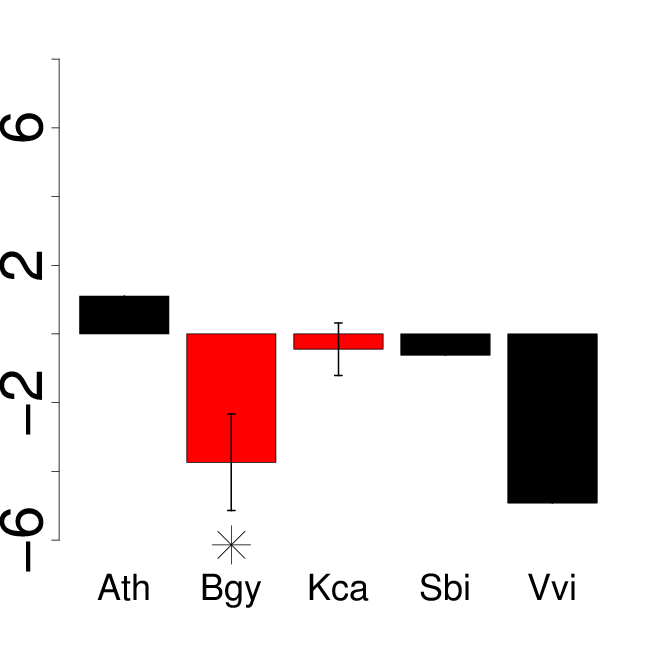

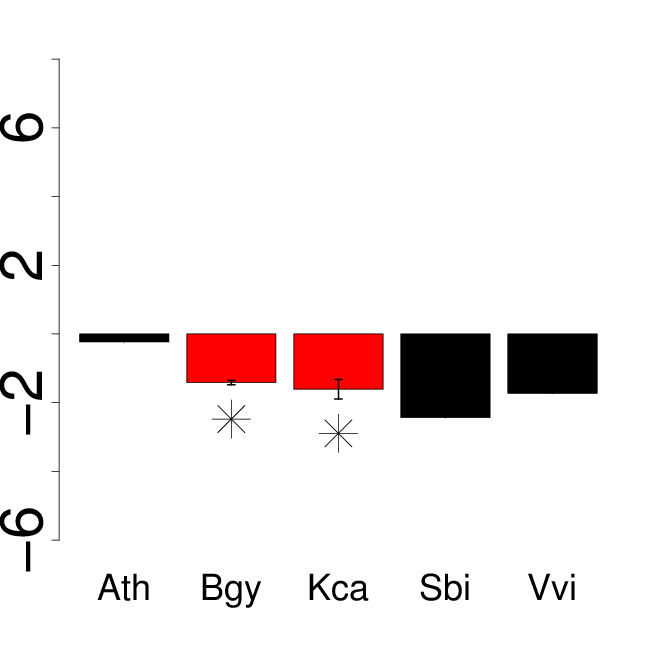


miR156 miR164 miR165/166 miR395 miR396


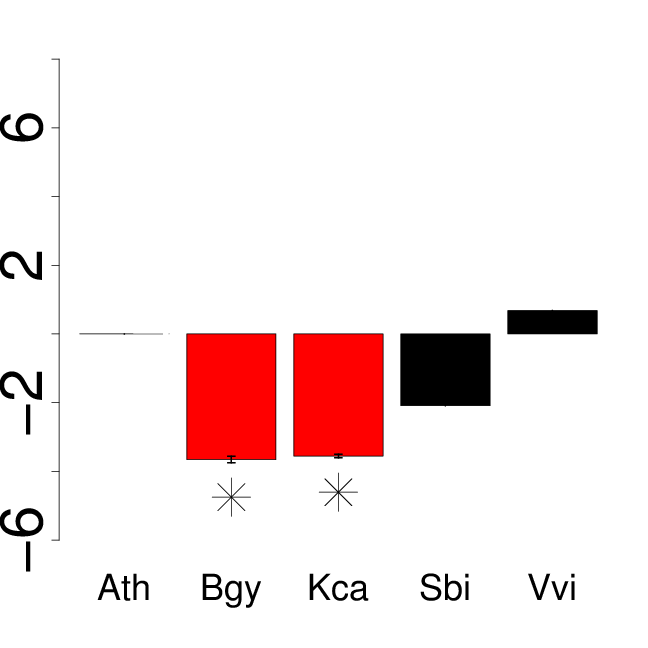

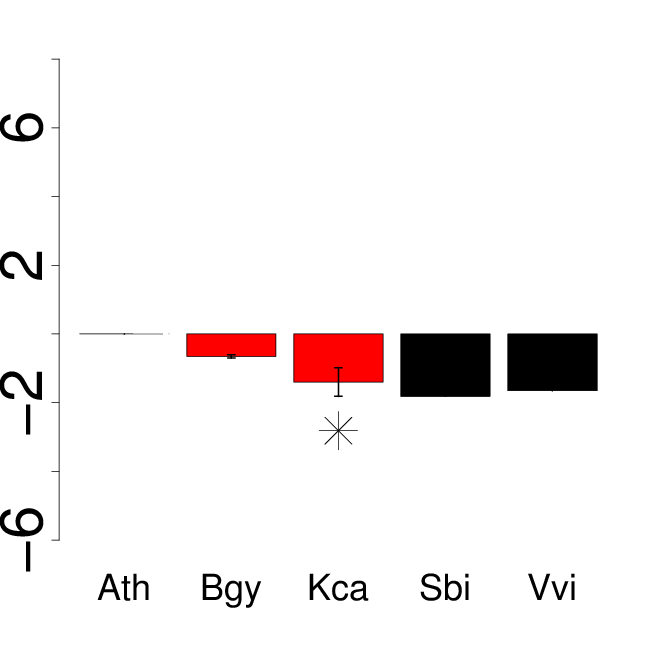

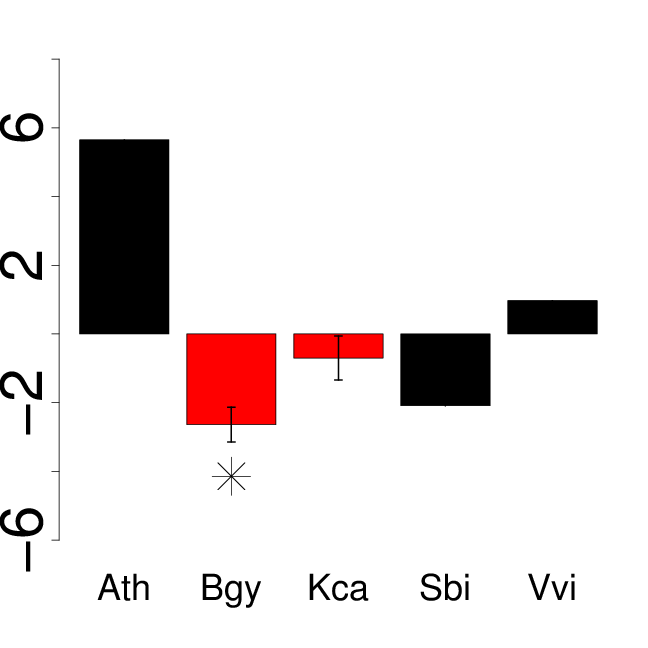

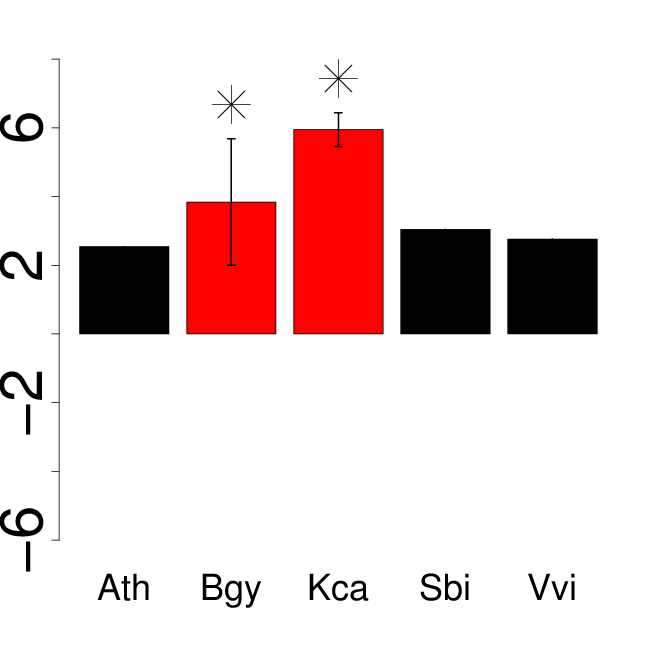

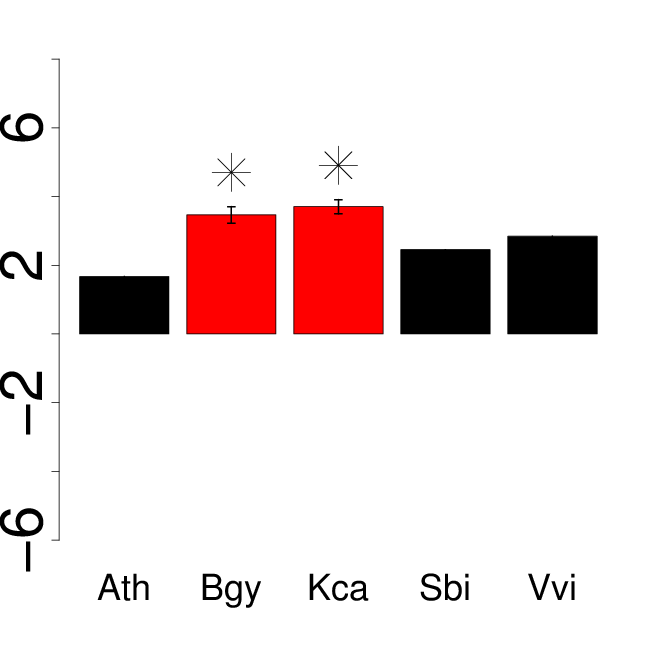


NA

NA

miR530 miR535 miR858 miR319 miR390

Fig. S5.

**Fig. S6. Quantitative stem-loop RT-PCR validation and expression analysis of conserved mangrove** **miRNAs.** Error bars indicate the standard deviation of three biological replicates. Significant differences at p-value <= 0.01and <=0.001 (t-test) between samples are indicated with “*” and “***”, respectively. Abbreviations: Bgy, *B. gymnorhiza*; Kca, *K. candel* and Ath, *A. thaliana*; NA, not available.

**
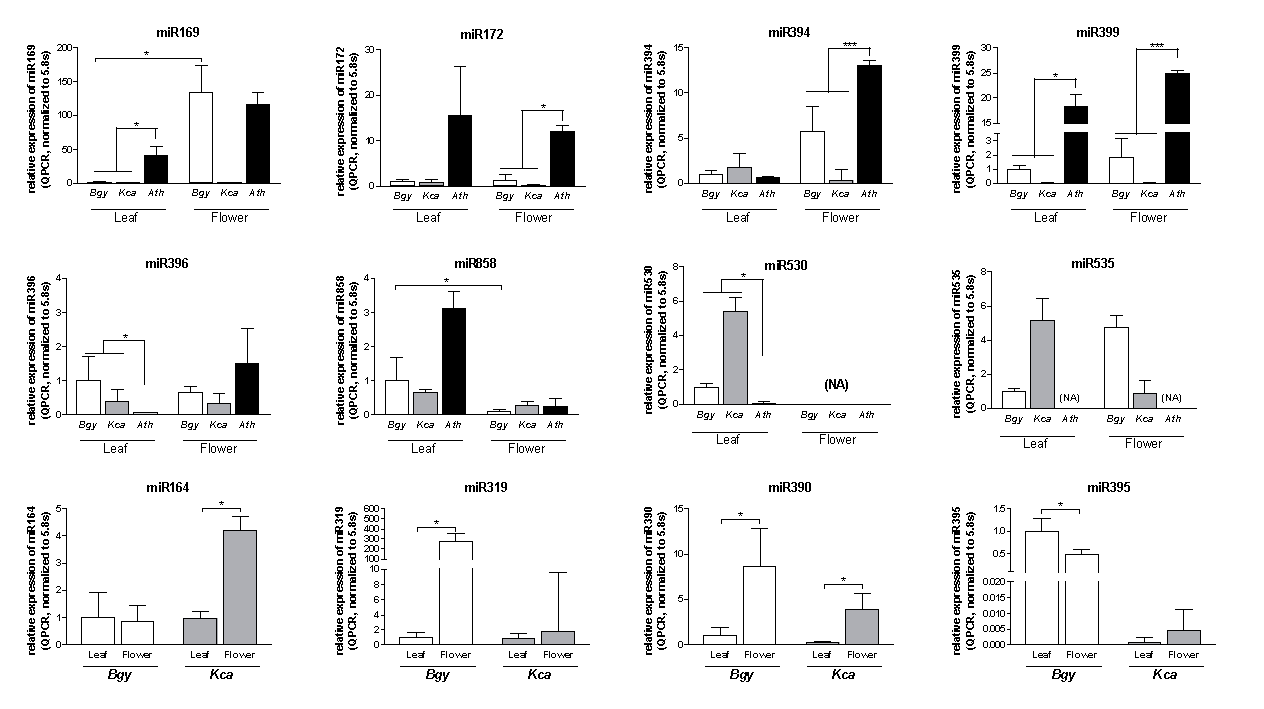
**

Fig. S6

**Fig. S7. Putative *TAS3* tasiRNAs in *B. gymnorhiza* libraries of leaves (a and c) and flower buds (b).** Vertical axis indicates the averaged abundance of 21-nt read counts along *BgTAS3* transcripts (BP947370)*.* Target site of miR390 is indicated with a bold arrow. Abbreviations: Bgy, *B. gymnorhiza*; F, flower buds; L, leaf; 1/2, biological repeats.

**a b**

**c**


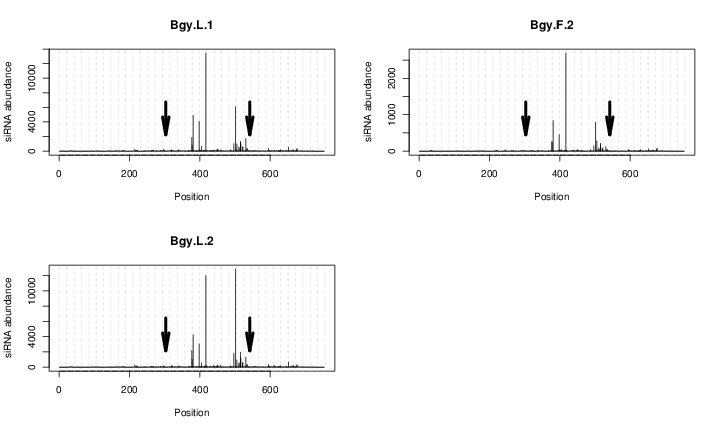


Fig. S7

**Fig. S8. Putative *TAS4* tasiRNAs in four *B. gymnorhiza* libraries of leaves (a and c) and flower buds (b and d).**Vertical axis indicates the averaged abundance of 21-nt read counts along *BgTAS4* transcripts (BP947370)*.* Target site of miR858 is indicated with a bold arrow and the four types of most abundant phased small RNAs are indicated with narrow arrows. Dash lines are plotted in a 21-nt phase. Abbreviations: Bgy, *B. gymnorhiza*; F, flower buds; L, leaf; 1/2, biological repeats.


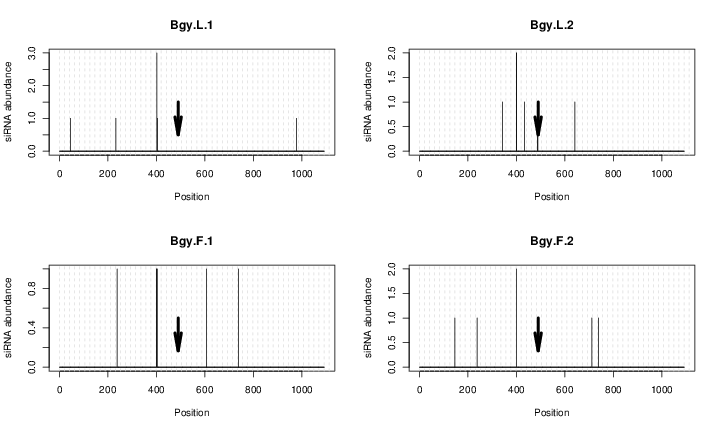


**a b**

**c d**

Fig. S8

**Table S1. Summary statistics for small RNA sequencing libraries.**

|  |  |  |  | |  |  |  | |
| --- | --- | --- | --- | --- | --- | --- | --- | --- |
|  |  | ***Flower buds*** | | |  | ***Leaf*** | | |
| **Species** | **Category** | **Redundant** | | **Non-redundant** |  | **Redundant** | | **Non-redundant** |
| ***Bruguiera***  ***gymnorhiza 1*** | Adaptor removed/Quality control/19-30 nt length | 24511652 | | 4745639 |  | 22509877 | | 4104628 |
|  | Non-coding RNAs^a^ removed | 21713516 | | 4566437 |  | 20632797 | | 3942450 |
|  | Repeat/Transposon^b^ removed | 21524881 | | 4522166 |  | 20453558 | | 3905236 |
|  | Known miRNAs^c^ removed | 18137099 | | 4514008 |  | 16883275 | | 3896100 |
|  | Novel miRNAs removed | 18136677 | | 4513950 |  | 16882977 | | 3896052 |
|  |  |  | |  |  |  | |  |
| ***Bruguiera***  ***gymnorhiza 2*** | Adaptor removed/Quality control/19-30 nt length | 23048687 | | 4316490 |  | 22905522 | | 4264720 |
|  | Non-coding RNAs^a^ removed | 19559843 | | 4146279 |  | 20789961 | | 4090193 |
|  | Repeat/Transposon^b^ removed | 19435436 | | 4114167 |  | 20559327 | | 4051663 |
|  | Known miRNAs^c^ removed | 15600881 | | 4106172 |  | 17756858 | | 4042624 |
|  | Novel miRNAs removed | 15600574 | | 4106124 |  | 17756473 | | 4042568 |
|  |  |  | |  |  |  | |  |
| ***Kandelia***  ***candel 1*** | Adaptor removed/Quality control/19-30 nt length | 22967675 | | 3537698 |  | 21428596 | | 1902662 |
|  | Non-coding RNAs^a^ removed | 21484276 | | 3406674 |  | 19129093 | | 1761990 |
|  | Repeat/Transposon^b^ removed | 21368979 | | 3380345 |  | 19008647 | | 1746924 |
|  | Known miRNAs^c^ removed | 13676446 | | 3370427 |  | 15259782 | | 1738081 |
|  | Novel miRNAs removed | 13676226 | | 3370398 |  | 15259437 | | 1738039 |
|  |  |  | |  |  |  | |  |
| ***Kandelia***  ***candel 2*** | Adaptor removed/Quality control/19-30 nt length | 21622810 | | 3137393 |  | 20718502 | | 1532139 |
|  | Non-coding RNAs^a^ removed | 19771955 | | 2999983 |  | 18208411 | | 1396622 |
|  | Repeat/Transposon^b^ removed | 19654754 | | 2975900 |  | 18118404 | | 1384223 |
|  | Known miRNAs^c^ removed | 13641320 | | 2966135 |  | 13601064 | | 1375574 |
|  | Novel miRNAs removed | 13641119 | | 2966102 |  | 13600743 | | 1375530 |

**Table S2 List of public small RNA datasets used in this study.**

| **Species** | **Tissues** | **Platform** | **Source** |
| --- | --- | --- | --- |
| *A. thaliana* | Leaf | Solexa | GSM738727^a^ |
| *A. thaliana* | Flower | Solexa | GSM738731^a^ |
| *P. trichocarpa* | Leaf | Solexa | http://smallrna.udel.edu/data-files/trimmed/PTR1_trim.zip |
| *V. vinifera* | Leaf | Solexa | http://smallrna.udel.edu/data-files/trimmed/VVI1_trim.zip |
| *V. vinifera* | Flower | Solexa | <http://smallrna.udel.edu/data-files/trimmed/VVI2_trim.zip> |
| *S. bicolor* | Leaf | Solexa | http://smallrna.udel.edu/data-files/trimmed/SBI1_trim.zip |
| *S. bicolor* | Flower | Solexa | <http://smallrna.udel.edu/data-files/trimmed/SBI2_trim.zip> |

**^a^** Data from reference ([Liu *et al.* 2012](#_ENREF_1))

**Table S3.** **Read counts and RPM for each homologous plant miRNAs. (XLS)**

**Table S4 Prediction and GO annotation for the targets of conserved and novel miRNA. (XLS)**

**Table S5 . Sequences of primers used in this study.**

|  | |
| --- | --- |
| Name ^a^ | Sequence (5'-3') |
| **RT-PCR of miRNAs** | |
| UPL_athmiR172 | GTTGGCTCTGGTGCAGGGTCCGAGGTATTCGCACCAGAGCCAACATGCAG |
| athmiR172_F | CGCCGAGAATCTTGATGATG |
| UPL_athmiR394 | GTTGGCTCTGGTGCAGGGTCCGAGGTATTCGCACCAGAGCCAACGGAGGT |
| athmiR394_F | CGCCGTTGGCATTCTGTCC |
| UPL_athmiR396 | GTTGGCTCTGGTGCAGGGTCCGAGGTATTCGCACCAGAGCCAACCAGTTC |
| athmiR396_F | CGCCGTTCCACAGCTTTCTT |
| UPL_athmiR399 | GTTGGCTCTGGTGCAGGGTCCGAGGTATTCGCACCAGAGCCAACCAGGGC |
| athmiR399_F | CGCCGTGCCAAAGGAGAGTT |
| UPL_athmiR530 | GTTGGCTCTGGTGCAGGGTCCGAGGTATTCGCACCAGAGCCAACAAGGTG |
| athmiR530_F | CGCCGTGCATTTGCACCTG |
| UPL_athmiR535 | GTTGGCTCTGGTGCAGGGTCCGAGGTATTCGCACCAGAGCCAACGCGTGC |
| athmiR535_F | CGCCGTGACAACGAGAGAGA |
| UPL_athmiR858b | GTTGGCTCTGGTGCAGGGTCCGAGGTATTCGCACCAGAGCCAACCAAGGT |
| athmiR858b_F | CGCCGTTCGTTGTCTGTTCG |
| UPL_athmiR169a | GTTGGCTCTGGTGCAGGGTCCGAGGTATTCGCACCAGAGCCAACTCGGCA |
| athmiR169a_F | CGCCGCAGCCAAGGATGACT |
| UPL_athmiR164a | GTTGGCTCTGGTGCAGGGTCCGAGGTATTCGCACCAGAGCCAACTGCACG |
| athmiR164a_F | CGCCGTGGAGAAGCAGGGCA |
| UPL_athmiR395a | GTTGGCTCTGGTGCAGGGTCCGAGGTATTCGCACCAGAGCCAACGAGTTC |
| athmiR395a_F | CGCCGCTGAAGTGTTTGGGG |
| UPL_athmiR319a | GTTGGCTCTGGTGCAGGGTCCGAGGTATTCGCACCAGAGCCAACAGGGAG |
| athmiR319a_F | CGCCGTTGGACTGAAGGGAG |
| UPL_athmiR390a | GTTGGCTCTGGTGCAGGGTCCGAGGTATTCGCACCAGAGCCAACGGCGCT |
| athmiR390a_F | CGCCGAAGCTCAGGAGGGAT |
| UPL_athmiR858a | GTTGGCTCTGGTGCAGGGTCCGAGGTATTCGCACCAGAGCCAACAAGGTC |
| athmiR858a_F | CGCCGTTTCGTTGTCTGTTC |
| UPL_5.8s rRNA RT | GTTGGCTCTGGTGCAGGGTCCGAGGTATTCGCACCAGAGCCAACGTGACG |
| 5.8s rRNA F | GCAGAATCCCGTGAACCATCG |
| Reverse | GTGCAGGGTCCGAGGT |
| **Primers for 5'RACE** | |
| BP945328-miR398R | TGCCCTCCCTTGCCCAGATCATCAG |
| BP952053-miR396R | CATGGTACCCCCGGCCGAGCA |
| Universal | CTAATACGACTCACTATAGGGCAAGCAGTGGTATCAACGCAGAGT |

^a^ F, forward; R reverse; RT, stem-loop RT

**Reference**

**Liu, C., M. J. Axtell, et al. 2012. The helicase and RNaseIIIa domains of Arabidopsis Dicer-Like1 modulate catalytic parameters during microRNA biogenesis. *Plant Physiol* 159(2): 748-758.**
